# Case Report: A Rare Case of Thrombotic Microangiopathy Induced by Remethylation Disorders

**DOI:** 10.3389/fmed.2022.837253

**Published:** 2022-03-02

**Authors:** Lu Pang, Jian Chen, Haiyan Yu, Haiming Huang, Bo Jin, Xin Wang, Haixia Li

**Affiliations:** ^1^Department of Clinical Laboratory, Peking University First Hospital, Beijing, China; ^2^Department of Pathology, National Cancer Center/National Clinical Research Center for Cancer/Cancer Hospital, Chinese Academy of Medical Sciences and Peking Union Medical College, Beijing, China

**Keywords:** hemolytic uremic syndrome, schistocytes, remethylation disorders, cblC, *MMACHC*

## Abstract

In this research, we described a very rare case of thrombotic microangiopathy induced by remethylation disorders. A 16-year-old boy presented to the emergency department with 5 months of weakness and fatigue. He was diagnosed with thrombotic microangiopathy based on clinical manifestation and laboratory information, which showed microangiopathic hemolytic anemia, renal impairment, and thrombocytopenia. After a complex diagnostic workup, the metabolite screening parameters and sequencing results guided us toward the diagnosis of remethylation disorders. The patient was diagnosed with thrombotic microangiopathy induced by remethylation disorders (cblC).

## Introduction

A hemolytic uremic syndrome is a form of thrombotic microangiopathy. Hemolytic uremic syndrome (HUS) encompasses several disorders: shiga toxin-induced HUS, and pneumococcus-induced HUS, HUS associated with complement dysregulation, HUS related to cobalamin C (cblC) defect, and HUS secondary to a heterogeneous group of causes (infections, drugs, cancer, and systemic diseases). Among these causes, cblC defect is one of the combined remethylation disorders with a genetically heterogeneous disorder of cbl metabolism. In this study, we presented a rare case with thrombotic microangiopathy (TMA) induced by remethylation disorders (cblC).

## Case Description

**Figure 1:**
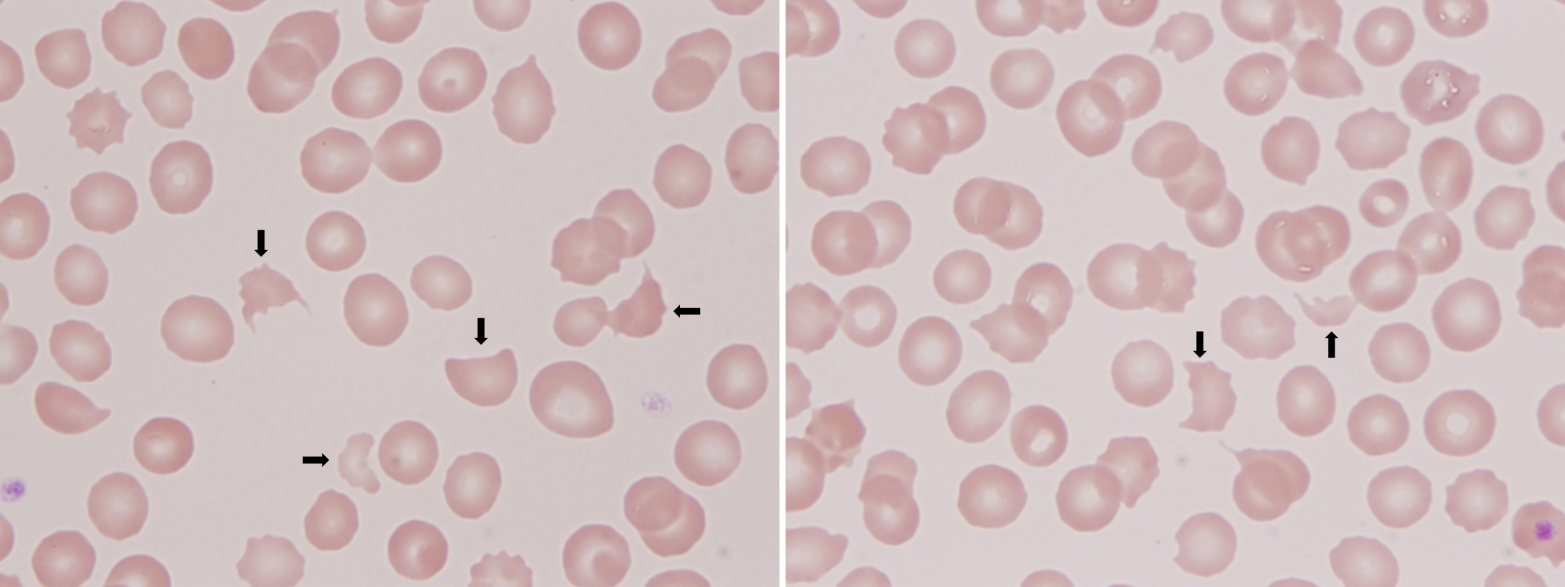
Wright-stained peripheral smear (PS) showed schistocytes. The arrows mean schistocytes.

**Figure 2:**
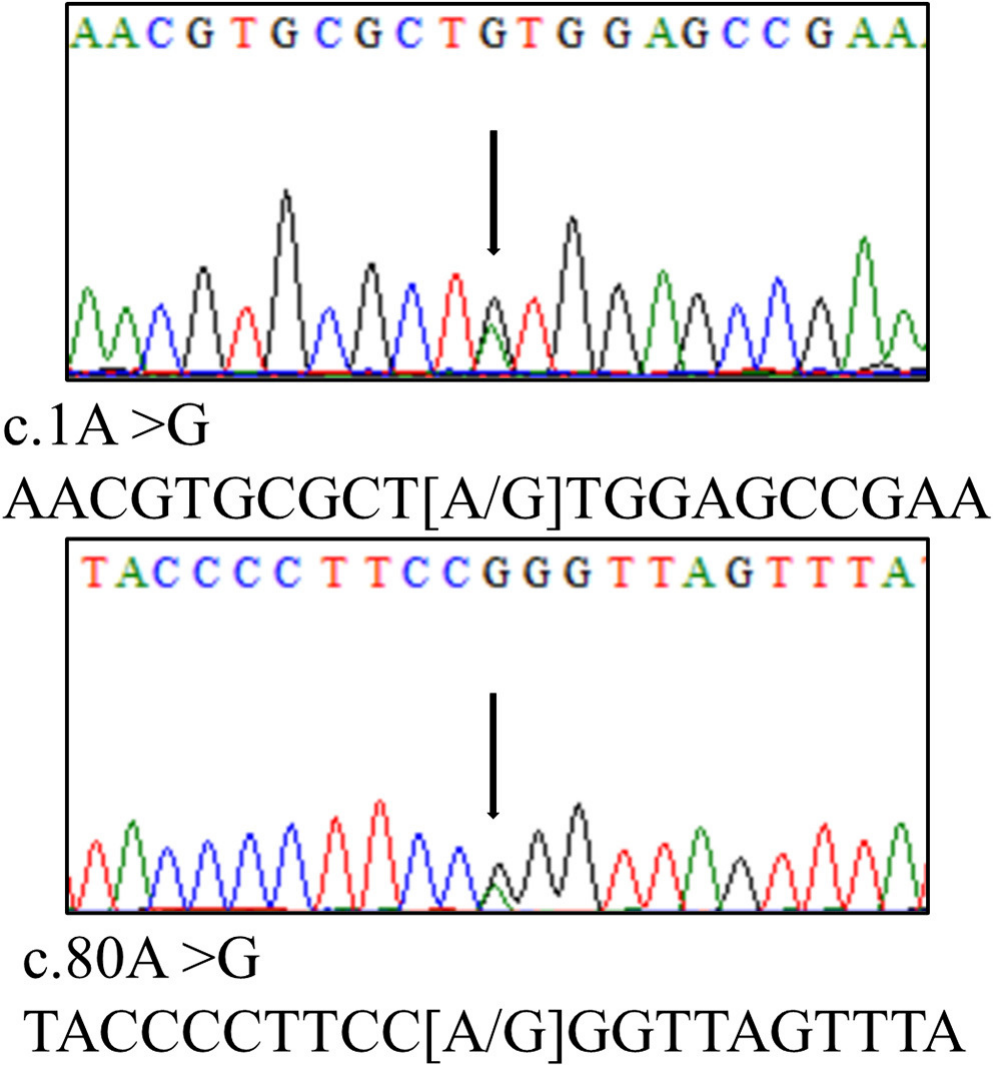
Genetic analysis revealed a compound heterozygous *MMACHC* mutation (c.1A > G and c.80A > G).

A 16-year-old boy presented to the emergency department with 5 months of weakness and fatigue. The patient was in good general health, had no relevant medical history, no abdominal pain, and diarrhea, and had a normal physical examination. Laboratory evaluation showed thrombocytopenia (platelet count, 58 × 10^9^/L) and hemolytic anemia (hemoglobin, 77 g/L; reticulocyte, 199.9 × 10^9^/L; lactate dehydrogenase, 609 IU/L). Additional laboratory tests showed an elevated creatinine level of 2.6 mg/dL and elevated 24 h proteinuria of 5.66 g/24 h. Serum vitamin B12 (348 nmol/L), folate (20.31 nmol/L), ferritin (169.2 ng/mL), iron (14.1 μmol/L), complement 3 (0.733 g/L), and complement 4 (0.325 g/L) were all within the normal range. Wright-stained peripheral smear (PS) showed schistocytes (Figure 1; original magnification × 1,000). Level of factor H and ADAMTS13 activity were normal. Anti-factor H-antibody and Coombs's test were negative. The autoantibodies, such as an anti-nuclear antibody, anti-dsDNA antibody, anti-phospholipase A2 receptor antibody, anti-phospholipid antibody, and anti-neutrophil cytoplasmic antibody were all negative. Renal pathology suggested that there were severe TMA with swelling endothelial cells, thickening arterial wall, thrombus in a capillary lumen, and IgM and C3 deposition. By screening for metabolic diseases, we found that the concentration of plasma total homocysteine (tHcy, 247.84 μmol/L), urinary methylmalonic acid (MMA, 62.29 mmol/mol creatinine), and blood propionyl carnitine (C3, 8.67 μmol/L) were increased obviously, and plasma methionine (Met, 7.8 μmol/L) was decreased. The whole exon sequencing and Sanger sequencing revealed a compound heterozygous *MMACHC* mutation (Figure 2; c.1A > G and c.80A > G).

The patient was diagnosed as TMA induced by remethylation disorders (cblC) according to the guideline (1). The patient was treated with betaine, hydroxycobalamin, and folic acid. After treatment, the tHcy level was normalized but hemolysis, thrombocytopenia, and acute kidney failure persisted. Because plasma exchange was ineffective, he required continued renal replacement treatment (CRRT) three times a week due to renal injury of TMA.

The study was in compliance with the Declaration of Helsinki and approved by the ethics committee of Peking University First Hospital. The significance of the research was explained to the patient and informed consent was obtained.

## Discussion

Remethylation disorders are rare inherited disorders in which impaired remethylation of homocysteine (Hcy) to Met. Met leads to accumulation of tHcy. Remethylation disorders can be classified into three groups (1). Combined remethylation disorders (cblC, cblD-MMA/HC, cblF, and cblJ) are alterations of the transport and intracellular metabolism of cobalamins, which causes a defect in the synthesis of the two functional forms of cobalamin (cbl; vitamin B12): methylcobalamin, cofactor of methionine synthase, and adenosylcobalamin, a cofactor for methylmalonyl-CoA mutase (MCM). Isolated remethylation disorders (cblD-HC, cblE, and cblG) are isolated deficits in the production of methylcobalamin. 5,10-methylenetetrahydrofolate reductase (MTHFR) deficiency is an abnormality of the folate cycle.

The clinical diagnosis of remethylation disorders can be easily and rapidly diagnosed by biochemical investigation. Serum vitamin B12, serum folate, plasma tHcy, plasma Met, blood acylcarnitine profile, plasma, or urinary MMA are required. Combined remethylation disorders are associated with increased tHcy and MMA (2). In this case, Serum Vitamin B12 and folate were within the normal range. The concentration of plasma tHcy, urinary MMA, and blood C3 were increased obviously. The plasma Met was decreased. Therefore, this case was diagnosed as a combined remethylation disorder.

Combined remethylation disorders are genetically heterogeneous disorders of cbl metabolism. Different forms of the disorder have been classified as cblC, cblD-MMA/HC, cblF, and cblJ. Among these, cblC type is most frequent and caused by a mutation in the *MMACHC* gene on chromosome 1p34.1. The three most common mutations are c.271dupA, c.394C > T, and c.331C > T, However, in Chinese patients, the c.609G > A mutation accounts for 85% of the identified alleles in *MMACHC* mutation (1). In this case, heterozygous mutations of *MMACHC* (c.1A > G and c.80A > G) were found, which have been reported before (3), although the mutations are uncommon in Chinese patients.

cblC defect usually occurs during the 1st year of life, and late-onset is applicable to children older than 4 years. There is great variability in the clinical presentation of cblC defect, and more common in the blood and nervous system (3). In this study, we focus on the TMA, which is a rarely late-onset manifestation in cblC defect.

Hemolytic uremic syndrome (HUS) is a form of TMA, characterized by microangiopathic hemolytic anemia, renal impairment, and thrombocytopenia (4). Another form of TMA is thrombotic thrombocytopenic purpura (TTP) defined by a severe deficiency in ADAMTS13, the specific von Willebrand factor (vWF)-cleaving protease (5). It is worth noting that schistocytes are fragments of red blood cells produced by extrinsic mechanical damage and a diagnostic feature of TMA (6). A percentage of schistocytes above 1% is a robust indicator for the diagnosis of TMA (7). In this case, the thrombocytopenia, hemolytic anemia, renal dysfunction, schistocytes, and normal ADAMTS13 activity all support the diagnosis of TMA.

The HUS encompasses a heterogeneous group of disorders, including typical HUS due to an infection from shiga toxin-producing Escherichia coli (STEC), and atypical hemolytic uremic syndrome (aHUS), caused by dysregulation of the alternative pathway of complement secondary to complement gene mutations or anti-complement factor H antibodies (8). cblC is a genetic disease that can lead to HUS (9). So far, only a few cases of HUS have been triggered by a metabolic disorder of cbl (10). Although HUS can be caused by cblC defect accompanied by complement dysregulation (11), dysregulation of the complement alternative pathway was not found with the whole-exome sequencing and ADAMTS13 test in this case. This patient is not complement-driven and, therefore, cannot benefit from complement inhibitors (12). Based on the diagnoses of cblC defect, the patient was diagnosed as TMA induced by combined remethylation disorders (cblC).

In this study, we presented a case of combined remethylation disorder, which is cblC defect and *MMACHC*-related, is the most common inborn error of cbl metabolism and it is a rare cause of TMA. Combined remethylation disorders should be considered in those patients with unclear microangiopathic hemolytic anemia. Therefore, plasma tHcy and MMA measurement should be included in the workup of renal TMA children. Plasma tHcy is readily available in clinical laboratories and easily ruled the suspicion of a cbl metabolism defect, so it is the first biochemical parameter to assess when a remethylation disorder is suspected. Thereafter, the combination of methylmalonic aciduria, homocystinuria, and normal serum vitamin B12 concentration distinguish patients with a genetic defect of Cbl from vitamin B12 deficiency due to nutritional deficiency or acquired malabsorption (1).

## Data Availability Statement

The original contributions presented in the study are included in the article/supplementary material, further inquiries can be directed to the corresponding author/s.

## Ethics Statement

The studies involving human participants were reviewed and approved by the Ethics Committee of Peking University First Hospital. Written informed consent to participate in this study was provided by the participants' legal guardian/next of kin.

## Author Contributions

LP, JC, and HL conceived the manuscript, performed analysis, and wrote the manuscript. HY, HH, BJ, XW, LP, and HL performed interpretation of clinical data and contributed to manuscript writing. All authors contributed to the article and approved the submitted version.

## Conflict of Interest

The authors declare that the research was conducted in the absence of any commercial or financial relationships that could be construed as a potential conflict of interest.

## Publisher's Note

All claims expressed in this article are solely those of the authors and do not necessarily represent those of their affiliated organizations, or those of the publisher, the editors and the reviewers. Any product that may be evaluated in this article, or claim that may be made by its manufacturer, is not guaranteed or endorsed by the publisher.

## Abbreviations

aHUS: atypical hemolytic uremic syndrome
C3: propionyl carnitine
cbl: cobalamin
HUS: hemolytic uremic syndrome
Met: methionine
MMA: methylmalonic acid
MTHFR, 5: 10-methylenetetrahydrofolate reductase
PS: peripheral smear
STEC: shiga toxin-producing Escherichia coli
tHcy: total homocysteine
TMA: thrombotic microangiopathy
TTP: thrombotic thrombocytopenic purpura
vWF: von Willebrand factor.
